# Navigating climate change: Climate change awareness and strategies in micro, small, and medium-sized enterprises in a developing economy

**DOI:** 10.1371/journal.pone.0327165

**Published:** 2025-07-02

**Authors:** Julián Andrés Díaz Tautiva, Sebastian Barros-Celume, Antonio Lecuna, Camila Barragan-Rodriguez

**Affiliations:** 1 Centro de Gestión y Economía Aplicada, Departamento de Ciencias Económicas y Administrativas, Facultad de Ciencias Jurídicas, Económicas y Administrativas, Universidad Católica de Temuco, Temuco, La Araucania, Chile; 2 SK Research, Oxford Business College, Oxford, United Kingdom; 3 Facultad de Ciencias Económicas y Administrativas, Universidad Católica de la Santísima Concepción, Concepción, Biobío, Chile; 4 Katz School of Business, Fort Lewis College, Durango, Colorado, United States of America; 5 Facultad de Economía y Negocios, Universidad Del Desarrollo, Santiago, Región Metropolitana, Chile; Tsinghua University, CHINA

## Abstract

Given the limited research on climate change (CC) awareness and strategies among micro, small, and medium-sized enterprises (MSMEs), this study aims to address three key questions: To what extent are MSMEs aware of CC and decarbonization? How do MSMEs strategize to address CC and achieve carbon neutrality? What factors shape MSMEs’ attention to CC and decarbonization? Drawing on theoretical insights from the Attention-Based View, we analyze CC awareness and strategies among 1,453 Colombian ventures to identify common patterns and traits between them. Our findings reveal a significant gap in MSMEs’ understanding of essential concepts such as carbon neutrality, decarbonization, and the overall impacts of CC on their businesses. Moreover, the results indicate that most MSMEs exhibit greater awareness of CC-related strategies at the corporate practice level than those aimed at operational processes. On average, respondents estimate that achieving carbon-neutral emissions will take between five to twenty years. Based on our findings, we propose scholarly, managerial, and policy recommendations to enhance CC awareness and promote effective CC-related strategies across MSMEs from different economic sectors.

## Introduction

Among industrialized economies, around 95 percent of organizations are categorized as Micro, Small, and Medium-Sized Enterprises (MSMEs) [1]. MSMEs are key to economic growth and development, producing more than 50 percent of the total value added and 60 percent of private sector jobs [2]. However, on a global scale, MSMEs contribute significantly to environmental degradation, accounting for approximately 50–60 percent of total carbon emissions worldwide [1,3,4]. Therefore, they highly contribute to the increasing effects of climate change (CC), which is understood as massive large discontinuous changes resulting in alterations in the global average temperature, affecting the capacity of natural ecosystems, generating disruptions in precipitation patterns, and increasing the occurrence of extreme weather events [2,5–7]. As a result, CC introduces uncertainty regarding the availability of production factors (e.g., energy, raw materials, and human capital), and disruptions in the business environment itself, which may impact organizational growth and survival [8–13].

Despite the crucial role of MSMEs in the rising effects of climate change, most research on organizational strategies related to climate change has focused on large corporations. Existing literature has extensively explored how large firms adapt their business operations [14] in response to increasing scrutiny from various stakeholders [15]. Extant research has also examined the organizational determinants of environmental strategies under uncertain conditions [16], the growing trend of disclosure among large firms to comply with evolving standards and regulations [17,18], and their cooperative and innovation-driven initiatives to mitigate environmental impacts and externalities [19]. Additionally, the literature has analyzed how contextual factors—such as normative, market, and social pressures—shape corporate environmental behavior, long-term investments, mitigation efforts, and economic outcomes [20–22]. Moreover, previous research has examined how some industries have shifted their processes, adopting cleaner production inputs [23,24].

Nonetheless, little empirical attention thus far has focused on how MSMEs perceive and strategize toward CC [25,26]. This gap may be partially explained by the weaker law enforcement regarding MSMEs’ environmental behavior compared to larger organizations, thus limiting available information for large empirical studies among MSMEs. This issue becomes increasingly prominent in developing markets [4,27], where environmental regulations targeting MSMEs are fewer and where official data among MSMEs is even scarcer and harder to find. As a result, research on MSMEs’ environmental practices remains inconclusive, inconsistent, and disjointed [3]. To face this concern, a body of literature has emerged in the last couple of years to understand the actions and decisions through which MSMEs develop a sustainability orientation [28,29] and their attitudes and strategic responses toward CC [30,31]. Overall, there is an increasing interest in understanding how MSMEs perceive CC and how public policies could help MSMEs address this grand global challenge [32].

Following the call toward guiding MSMEs’ actions towards a greener economy [26], this research aims to answer three interrelated questions: (a) To what extent are MSMEs aware of CC and decarbonization? (b) How do MSMEs develop strategies to address CC and achieve carbon neutrality? (c) What factors shape MSMEs’ attention to CC and decarbonization? To explore these questions, we analyzed the awareness and strategic approaches of 1,453 ventures in Colombia—a developing economy in the Global South— specifically focusing on their awareness of CC and carbon neutrality. Our results indicate a significant heterogeneity in awareness levels influenced by economic sectors. This variation in awareness shapes the strategic actions in response to CC-related pressures. Notably, while many MSMEs in our sample exhibited limited awareness of CC, most acknowledged an internal impact of CC on their operations, recognizing its direct effects on their business activities and outcomes. Furthermore, our findings suggest that MSMEs displayed greater awareness of strategies at the corporate practice level compared to those targeting operational processes. By analyzing and comparing MSMEs from different economic sectors, we gain granularity in understanding their commonalities and differences across a wide range of CC-related variables and dimensions. Based on this, we propose new avenues for research with implications for scholars, policymakers, and practitioners.

This article is structured as follows. First, section 2 presents the theoretical framework, providing a comprehensive literature review on the emergent topic of CC among MSMEs. Next, section 3 describes the material and methods. Afterward, section 4 presents the analysis of MSMEs’ perceptions, awareness, and CC strategies. Next, section 5 elaborates on the main implications of our findings. Finally, section 6 addresses the main limitations and concluding remarks.

## Literature review

### Climate change awareness and strategies in MSMEs

The literature linking MSMEs’ awareness and strategies toward CC is still in its infancy. Two primary streams have emerged to explore this phenomenon. The first stream investigates the determinants influencing organizational response to climate-related challenges [22,26,29,30,32–35]. Within this stream, Mungai, Ndiritu, and Rajwani (2023) [34] explored the drivers of environmentally driven policies and practices, finding that organizations that undergo an audit process and engage in internal awareness initiatives—such as compensation policies linked to environmental impact—experience improved environmental performance in terms of energy consumption and savings. In turn, Cai et al. (2022) [35] examine how external pressures foster awareness about climate change (CC). In short, they found that factors such as media influence and local corporate social responsibility initiatives significantly correlate with low-carbon behaviors among entrepreneurial ventures, thus, suggesting that external pressures can drive a shift toward sustainable practices. Similarly, Lo, Liu, and Cheung (2019) [33] explored how the attributes and capabilities of key corporate decision-makers shape awareness of CC risks. Overall, they find that executives operating in disadvantaged contexts—characterized by limited human and social capital—are more likely to recognize the vulnerability of their firms to extreme weather events. Likewise, Chowdhury, Maiti, and Bhattacharyya (2016) [32] explored CC awareness at the community level, noting that bridging the gap between scientific knowledge and practical application demands the development of multidimensional educational programs tailored to diverse target audiences. Importantly, Afolabi et al. (2023) [26] explored sustainable behavior among MSMEs, noting that their organizational objectives are often disconnected from greenhouse gas reduction goals. This misalignment is attributed to unclear sustainability targets, knowledge gaps about CC, a lack of perceived benefits from addressing environmental concerns, and limited information on sustainability reporting and best practices. Finally, Fuchs, Mohan, and Strob (2023) [29] investigated awareness patterns among Swiss MSMEs, finding that firms with limited human capital exhibit lower CC awareness than larger corporations. Ultimately, their findings reinforce that awareness is closely tied to the environmental knowledge resources within each firm.

The second research stream delves into the determinants of CC strategies [25,27,28,36–39]. For instance, Dale et al. (2022) [27] proposed that MSMEs’ strategic choices are heavily influenced by their capacity to adapt by selecting suitable management tools to address immediate and long-term environmental risks. Similarly, Luederitz et al. (2021) [28] highlighted that MSMEs develop sustainability strategies based on perceived value creation across environmental, social, and economic domains, often shaped through multi-stakeholder dialogues and influenced by the experiences and perceptions of organizational members. In turn, Sáez-Martínez, Díaz-García, and González-Moreno (2016) [37] found that external pressures, such as market demands and regulatory requirements, trigger sustainability strategies across firms. Henriques and Catarino (2016) [36] found that energy-related decisions depend on firm-specific characteristics, such as information accessibility and cognitive resources. Lastly, Nejati, Amran, and Ahmad (2014) [25] suggested that firms experiencing pressures from employees and customers tend to adopt more proactive environmental practices, like reducing greenhouse gas emissions. This implies that both external pressures and internal resources play a significant role in shaping CC strategies among MSMEs.

### Theoretical insights: Attention-based view

Concerning organizational awareness and responses to CC, we draw theoretical insights from the Attention-Based View (ABV) [15,40–42]. In brief, ABV posits that organizations prioritize their attention—essentially their awareness—based on the most pressing issues impacting their organizational goals and survival [40,42]. Specifically, organizations notice, encode, interpret, and concentrate their time and effort on specific environmental concerns and strategies linked to them [42]. As such, organizations recognize contextual factors as key drivers for understanding and assessing their vulnerability to CC [41]. Indeed, organizations allocate their attention and resources to strategies that integrate sustainability objectives with operational goals, ensuring alignment between environmental and business imperatives, thus, responding selectively to ecological pressures and normative discourses [42]. However, firms that perceive CC as a low-priority issue tend to focus solely on financial value creation, prioritizing short-term profitability over sustainability. Importantly, perceived ecological pressures—driven by factors such as carbon market availability, the nature of the business community, and the economic sector—along with normative discourses—shaped by external pressures—may constrain firms’ strategic choices and attention toward sustainability-oriented strategies and practices.

## Data and methods

### Research paradigm and context

Given the complexity and multidimensional nature of CC’s impact on business activities, this study adopts a pragmatic philosophical approach. This perspective, rooted in non-reductive naturalism, emphasizes problem-solving and practical knowledge above other considerations [43,44]. In essence, a pragmatic philosophical perspective focuses on how our social world continually unfolds through collective efforts to address the demands of modern life [44]. Such an approach highlights the importance of aligning beliefs with actions in the inquiry process that underpins the pursuit of new knowledge [43]. From this standpoint, research endeavors aim to develop novel and useful insights about phenomena, enhancing human perspectives, beliefs, and experiences [45].

Although existing research has emphasized the influence of contextual characteristics on MSMEs’ awareness and strategies toward CC [3], the majority of studies have focused on MSMEs within African [34,38], Asian [25,27,31,32,35], and European contexts [14,26,30,36,37,39,46], leaving the Americas relatively unexplored [3,7]. To address this gap, we conducted an exploratory, deductive quantitative study on Colombian MSMEs, aiming to deepen and expand the contextual understanding of CC awareness and strategic responses among these ventures within a developing Latin American country. Latin America has been widely conceptualized as a fertile context for theory building and empirical testing in the management field [47]. Colombia, as a developing nation and signatory to key international climate agreements—including the United Nations Framework Convention on Climate Change (1992), the Kyoto Protocol (1998), and the Paris Agreement (2015)—has committed to ambitious climate goals. These include a 51 percent reduction in greenhouse gas emissions by 2030 and the achievement of carbon neutrality by 2050. To this end, Colombia has established industry-specific targets under Law 2169/2021, focusing on sectors such as mining and quarrying, tourism, and manufacturing.

For this purpose, since 2016 Colombia has pursued a continual legislative agenda to reduce CC [48,49]. Specifically, it has ratified several green tax laws targeting greenhouse emissions from producers and importers of fossil fuels (Law 1819/2016). In addition, the Colombian government has imposed a specific tax for each ton of greenhouse emissions (i.e., 25,799.56 Colombian pesos- approximately 6.37 United States Dollars- in 2024) for highly polluting and resource-intensive industries. More so, several Colombian governments have established general CC regulations for large firms, financial corporations, and firms within the energy sector (Law 1931/2018). These regulations compel enterprises to elaborate emission reports, mitigation and adaptation plans, and environmental responsibility strategies, establishing various economic incentives to reduce greenhouse emissions.

**Fig 1:**
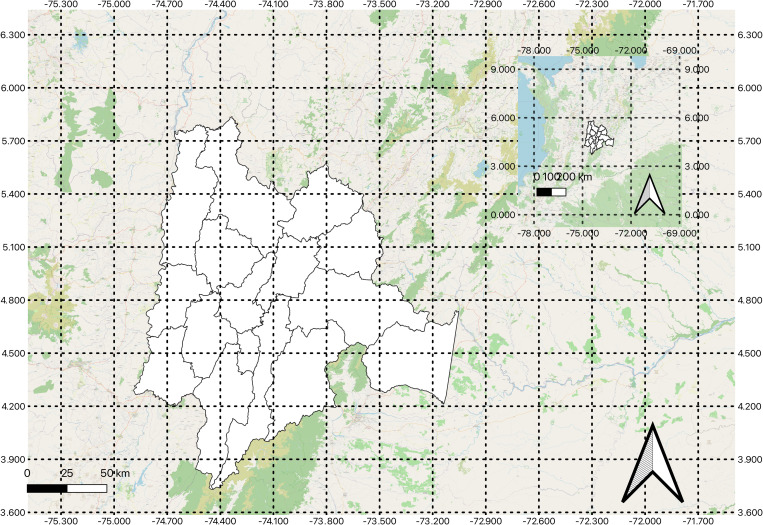
Geographical location of the Cundinamarca Region and the research site.

Fig 1 illustrates the geographical scope of our research, which falls under the administrative jurisdiction of the largest business association in Colombia. This meta-organization oversees nine provinces within the Cundinamarca region: Bogotá, Sumapaz, Soacha, Oriente, Sabana Centro, Guavio, Medina, Almeidas, and Ubaté. These provinces encompass 59 municipalities and the capital city, forming a business community of 495,981 active ventures as of 2023 [50]—representing approximately 28.5 percent of Colombia’s active businesses. Of these, 85 percent are concentrated in the capital, while 15 percent are distributed across the municipalities. Overall, MSMEs account for 99.3 percent of all ventures in the region. In 2023, this region contributed approximately 31.3 percent of the national Gross Domestic Product, generating around 4.1 percent of Colombia’s total carbon emissions (approximately 9.91 million tons of greenhouse gases), with nearly 15 percent of these emissions originating from ventures in the secondary economic sector [51]. Consequently, this contextual setting provides a robust foundation for addressing our research questions and objectives.

### Sample and survey

We partnered with the largest business association in Colombia to analyze firm-level data collected through a phone survey conducted as part of the Industrial Decarbonization Project. The anonymized dataset was provided to the research team in February 2024. An independent company surveyed between May and August 2023, employing a stratified probabilistic sampling method to ensure a 5 percent margin of error and a 95 percent confidence level. The survey targeted key respondents within the firms—business owners, C-level executives, and sustainability managers—possessing relevant knowledge of environmental and sustainability-related organizational strategies and practices. Data collection was conducted via phone interviews using a structured questionnaire. Respondents provided verbal informed consent, which was recorded. The selection of this survey method was based on three interrelated factors: (1) an increased likelihood of response from key participants compared to web-based surveys, considering the cultural characteristics of Colombian respondents; (2) budget constraints associated with implementing the Industrial Decarbonization Project; and (3) the opportunity to clarify complex concepts in real time if respondents had doubts. Previous research highlights the advantages of this sampling approach, emphasizing its accessibility across diverse geographical and individual contexts and the potential for respondents to feel more comfortable describing their perspectives compared to face-to-face or web-based surveys [52]. The data collection process strictly adhered to Colombian data protection regulations, including Law 1581 of 2012 and Law 1266 of 2008, and complied with the ethical principles outlined in the Declaration of Helsinki.

The survey focused on three core components. First, firmographic data for each respondent, including the type of economic activity—whether the respondent was a juridical entity (i.e., formal organizations registered under Colombian business laws) or a natural person engaged in formal entrepreneurial activities. Additionally, information was collected on the economic sector, industry, and organizational size (measured by sales for juridical entities and by the number of employees for natural persons). Second, respondents provided insights on their knowledge and perceptions of CC and decarbonization, particularly their awareness of specific CC-related concepts. Respondents were also asked to identify entities within their value chain perceived to be at risk due to CC. Further questions focused on their awareness of CC strategies, distinguishing between those endorsed at the corporate level and those applied operationally. Finally, respondents shared their perceptions and awareness regarding carbon neutrality within their ventures. This section comprised three elements: the timeline in which they anticipated achieving carbon neutrality; their awareness of strategies at both corporate and operational levels aimed at attaining this sustainability goal and whether they had implemented any carbon-related practices or programs, and; lastly, factors that might influence their awareness of carbon neutrality, including leadership diversity, perceived pressure from key stakeholders (such as customers and investors), and awareness of the carbon market at the national level.

### Variables and measures

In line with our research paradigm, we focused on policy-relevant variables related to CC and carbon neutrality. First, we conceptualized CC awareness as respondents’ perceived knowledge of key concepts, including “Global Warming,” “Climate Change,” “Greenhouse Gas Mitigation,” “Adaptation to Climate Change Impact,” “Offsetting of Greenhouse Gas Emissions,” “Carbon Neutrality,” and “Decarbonization.” Respondents rated their knowledge of these concepts using a 5-point Likert scale, ranging from “Low Knowledge” to “High Knowledge.” Additionally, we measured perceived risk associated with CC based on three potential impacts: direct impact on the firm itself, external impact on the supply chain (both on suppliers and customers), or no impact from CC at all. Additionally, we assessed strategic awareness of CC by examining whether respondents were knowledgeable about strategies at both the corporate and operational levels. At the corporate practice level, we considered awareness of practices such as using sustainable transportation (e.g., electric or hybrid vehicles), analyzing and measuring carbon footprint indicators, engaging in voluntary tree planting and reforestation, purchasing or issuing green bonds, and producing green energy. At the operational process level, we examined awareness of energy reduction and efficiency programs, environmental policy implementation, networking with clusters and associations focused on CC, adopting hybrid work models to reduce carbon emissions, green planting initiatives, and waste reclassification practices.

In addition to assessing strategic awareness, we identified the anticipated timeline for ventures to achieve carbon neutrality. Respondents were asked to choose from several options: within 5 years, between 6 to 10 years, between 11 to 20 years, more than 20 years, not in the company’s interest, deemed infeasible, or unknown. We also evaluated knowledge of specific carbon-neutral strategies at both the corporate and operational levels. At the corporate practice level, we considered strategies such as compensating for carbon emissions through reforestation or biodiversity restoration, implementing shared transport systems for employees, purchasing green bonds, and establishing “life fences” to enhance biodiversity. At the operational process level, we focused on practices like conducting environmental diagnostics of corporate impacts, optimizing the use of raw materials, providing training in environmental and energy management, adopting clean energy sources, and implementing comprehensive waste management systems. To further analyze the factors influencing ventures’ awareness toward carbon neutrality, we incorporated several elements that describe external pressures and normative discourses shaping sustainability-oriented decisions. First, we included a dummy variable to assess whether organizational decisions were made by a gender-balanced executive team (i.e., 50 percent male, 50 percent female at the C-level), assigning a value of 1 if gender-balanced leadership was reported. Additionally, two dummy variables captured perceived pressures from key stakeholders: one indicating whether customer pressures were perceived as relevant or very relevant, and another for investor pressures. Each variable was assigned a value of 1 if the venture perceived these pressures as relevant or very relevant.

Furthermore, we complemented our analysis with two dummy variables measuring perceived support from the government and key stakeholders (employees). A value of 1 was assigned if the venture perceived these sources of support as relevant or very relevant. Additionally, we considered internal constraints by incorporating dummy variables reflecting capital (i.e., financial, human, or time) and technological barriers. Each variable was assigned a value of 1 if the venture identified these barriers as relevant. Lastly, we created three dummy variables to capture ventures’ awareness and engagement with carbon neutrality initiatives: (1) knowledge of carbon markets, assigned a value of 1 if the venture reported awareness of such markets; (2) involvement in carbon-neutral practices, assigned a value of 1 if the venture actively engaged in these practices; and (3) participation in organizational programs related to carbon neutrality, assigned a value of 1 if the venture was involved in such programs.

### Data analysis

Our data analysis consisted of three interrelated steps. First, we employed descriptive analysis techniques to identify common patterns regarding ventures’ awareness of CC and their strategic decisions toward carbon neutrality. Specifically, we categorized ventures into different subsamples based on organizational type and economic sector, focusing on comparing means across these subgroups. This descriptive analysis aimed to highlight variations and commonalities to uncover potential awareness gaps and shortcomings that could guide future research and the development of targeted public policies and interventions [27,36,38,53]. Subsequently, for each category of analysis, we conducted a non-parametric Pearson chi-square ($X^{2}$) test of homogeneity [54] to assess the distributional similarities within subgroups. This test enabled us to determine whether two or more populations shared similar distributions across a single categorical variable. We performed each test using the ‘*chisq.test’* function from the ‘*stats’* package in R, applied to count-based contingency tables. For each test we computed the p-value based on the asymptotic chi-squared distribution of the test statistic. The code and data supporting these analyses are publicly accessible via the DOI provided in the Data Availability Statement.

Lastly, we examined the factors influencing ventures’ awareness regarding (a) the feasibility of achieving carbon neutrality within five years and (b) whether carbon neutrality was perceived as unfeasible or not part of their strategic plan. To analyze these decisions, we employed a Probit model with robust standard errors. This model incorporated independent variables related to awareness, including capital and technology barriers, gender balance in leadership, perceived pressures from customers and investors, perceived support from the government and key stakeholders (employees), ventures’ awareness of carbon markets, and the adoption of carbon-related practices and programs. To mitigate potential omitted variable bias, we controlled for economic sector and organizational characteristics. The code and data supporting these analyses are publicly accessible via the DOI provided in the Data Availability Statement.

## Results

### MSMEs description

Initially, we explored the nature of the sample obtained through the survey, categorizing each respondent based on their nature (whether juridic or natural person). Given the scarcity of empirical studies exploring the perceptions, awareness, and CC strategies among non-large organizations, we developed three categories that encompass the majority of Colombian business ventures [55,56]: (a) Self-employed -SE- (i.e., single natural individuals who manage all business functions independently, that is, without employees); (b) Small Business Owners -SBO- (i.e., single natural individuals with at most ten employees but lack a formal organizational structure); and (c) Micro, Small, and Medium-sized Enterprises -MSMEs- (i.e., juridical persons with a formal organizational structure). These categories were chosen to capture different types of entities within the Colombian business community, allowing for a more nuanced analysis of the MSMEs’ CC awareness and strategic responses with relevant contrasting groups. Table 1 overviews the sample distribution categorized by the nature of the respondents and their corresponding economic classification (by economic sector and industry). Most of the sample consisted of SE (44.6 percent; N = 648), followed by SBO (37.9 percent; N = 551) and MSMEs (17.5 percent; N = 254). In terms of economic sectors, most of the sample was categorized within the tertiary economic sector (64.3 percent; N = 934), followed by the secondary sector (29.6 percent; N = 430) and the primary sector (6.1 percent; N = 89). Regarding industries, most of the sample consisted of ventures in the wholesale and retail trade industry (Tertiary Sector; 35.2 percent; N = 512), followed by manufacturing industries (Secondary Sector; 26.1 percent; N = 379) and accommodation and food services (Secondary Sector; 6.2 percent; N = 90). Overall, the sample distribution is representative of the research context under study, as it reflects the predominant economic activities within such, emphasizing the significant presence of the tertiary sector over the other ones.

**Table 1:** Sample Description.

Sectors By Economic classification	SE	SBO	MSMEs
**Grand Total**	254	648	551
**Primary Sector**	8	30	51
**Agriculture, Livestock, Hunting, Forestry, and Fishing**	6	23	38
**Mining and Quarrying**	1	1	9
**Water Distribution; Sewage and Wastewater Treatment,** **Waste Management, and Environmental Sanitation Activities**	1	6	4
**Secondary Sector**	36	170	224
**Construction**	1	12	37
**Electricity, Gas, Steam, and Air Conditioning Supply**	0	0	1
**Manufacturing Industries**	35	158	186
**Tertiary Sector**	210	448	276
**Accommodation and Food Services**	22	61	7
**Administrative and Support Service Activities**	8	15	19
**Artistic, Entertainment, and Recreation Activities**	0	8	4
**Education**	2	6	11
**Financial and Insurance Activities**	4	2	7
**Human Health and Social Work Activities**	2	6	4
**Information and Communications**	5	8	23
**Other Service Activities**	10	26	3
**Professional, Scientific, and Technical Activities**	11	24	42
**Public Administration and Defense; Compulsory Social Security**	0	0	1
**Real Estate Activities**	0	7	15
**Transportation and Storage**	2	10	16
**Wholesale and Retail Trade**	135	255	122
**Other commercial activities**	9	19	2
**Capital investors**	0	1	0

Note. SBO = Small Business Owners; SE = Self-Employed; MSMEs = Micro, Small, and Medium-sized Enterprises.

### Awareness: From global warming to decarbonization

Subsequently, we explored the awareness-perceived knowledge of the sample regarding CC and decarbonization (please refer to Table 2 for an overview). Panel A of Table II summarizes the results for the overall sample. The findings indicate that ventures show a considerable familiarity with general concepts such as “Global warming” (36 percent of the sample selected “5” on the scale; ${Mean\;}_{All}=3.79$) and “Climate Change” (36 percent of the sample selected “5” on the scale; ${Mean\;}_{All}=3.81$). However, knowledge levels are more moderate for terms such as “Greenhouse Gas Mitigation” (25 percent of the sample selected “3” on the scale; ${Mean\;}_{All}=2.82$), “Adaptation to climate change impacts” (29 percent of the sample selected “3” on the scale; ${Mean\;}_{All}=3.31$), and “Offsetting of greenhouse gas emissions” (25 percent of the sample selected “3” on the scale; ${Mean\;}_{All}=2.70$). In contrast, a significant portion of respondents was unfamiliar with “Carbon Neutrality” (38 percent of the sample selected “1” on the scale; ${Mean\;}_{All}=2.33$) and “Decarbonization” (33 percent of the sample selected “1” on the scale; ${Mean\;}_{All}=2.57$). These findings indicate that while there exists a slight common knowledge of general CC phenomena, more specific terms related to carbon management remain largely unknown among most respondents.

**Table 2:** Awareness and Perceived Knowledge.

Panel A. All sample								
Concept	1	2	3	4	5	Total	Mean	N
**1**	6.68	6.26	23.74	27.60	35.72	100.00	3.79	1,453
**2**	6.54	6.19	22.57	28.63	36.06	100.00	3.81	1,453
**3**	25.46	15.69	24.91	19.13	14.80	100.00	2.82	1,453
**4**	12.11	12.04	29.18	25.60	21.06	100.00	3.31	1,453
**5**	28.49	16.79	24.98	16.17	13.56	100.00	2.70	1,453
**6**	38.47	18.72	22.85	10.81	9.15	100.00	2.33	1,453
**7**	33.10	16.66	22.71	15.07	12.46	100.00	2.57	1,453
**Panel B. MSMEs**								
**Concept**	**1**	**2**	**3**	**4**	**5**	**Total**	**Mean**	**N**
**1**	4.90	3.81	21.05	30.13	40.11	100.00	3.97	551
**2**	5.44	4.54	17.79	31.58	40.65	100.00	3.97	551
**3**	20.15	12.16	25.05	23.77	18.87	100.00	3.09	551
**4**	9.98	10.53	24.32	31.58	23.59	100.00	3.48	551
**5**	22.50	15.79	22.69	21.78	17.24	100.00	2.95	551
**6**	32.67	16.88	23.96	13.79	12.70	100.00	2.57	551
**7**	28.86	15.61	21.05	17.60	16.88	100.00	2.78	551
**Panel C. SBO**								
**Concept**	**1**	**2**	**3**	**4**	**5**	**Total**	**Mean**	**N**
**1**	6.33	8.49	26.23	26.85	32.10	100.00	3.70	648
**2**	6.48	6.94	25.46	27.62	33.49	100.00	3.75	648
**3**	27.62	18.98	25.31	17.13	10.96	100.00	2.65	648
**4**	12.35	12.50	33.33	22.38	19.44	100.00	3.24	648
**5**	31.79	17.59	27.01	13.58	10.03	100.00	2.52	648
**6**	40.74	20.37	23.15	9.26	6.48	100.00	2.20	648
**7**	35.03	17.44	23.46	14.97	9.10	100.00	2.46	648
**Panel D. SE**								
**Concept**	**1**	**2**	**3**	**4**	**5**	**Total**	**Mean**	**N**
**1**	11.42	5.91	23.23	24.02	35.43	100.00	3.66	254
**2**	9.06	7.87	25.59	24.80	32.68	100.00	3.64	254
**3**	31.50	14.96	23.62	14.17	15.75	100.00	2.68	254
**4**	16.14	14.17	29.13	20.87	19.69	100.00	3.14	254
**5**	33.07	16.93	24.80	10.63	14.57	100.00	2.57	254
**6**	45.28	18.50	19.69	8.27	8.27	100.00	2.16	254
**7**	37.40	16.93	24.41	9.84	11.42	100.00	2.41	254

Note. Values in percentage. Concepts denote (1) Global warming; (2) Climate change; (3) Greenhouse gas mitigation; (4) Adaptation to climate change impacts; (5) Offsetting of greenhouse gas emissions; (6) Carbon neutrality (zero balance between emissions and offsets); (7) Decarbonization (having a low-emission production process). N = Sample Size. 5-point Likert scale where 1 indicates “Low Knowledge” and 5 indicates “High Knowledge”. SBO = Small Business Owners; SE = Self-Employed; MSMEs = Micro, Small, and Medium-sized Enterprises.

Furthermore, Panel B, C, and D in Table 2 present the results according to each category. There is a persistent trend across all types of ventures showing “High Knowledge” about concepts such as “Climate Change” and “Global Warming.” Specifically, MSMEs exhibit a higher awareness level (${Mean\;}_{MSMEs}=3.97$) in contrast to the other subsamples (${Mean\;}_{SE}=3.65$; ${Mean\;}_{SBO}=3.73$). Moreover, the MSMEs subsample showcases a higher awareness level regarding concepts such as “Greenhouse Gas Mitigation,” “Adaptation to Climate Change Impacts,” and “Offsetting of Greenhouse Gas Emissions” (${Mean\;}_{MSME}=3.17$) compared to the other subsamples (${Mean\;}_{SBO}=2.80$; ${Mean\;}_{SE}=\;2.80)$. Finally, the SE subsample shows the lowest awareness level about “Carbon Neutrality” and “Decarbonization” (${Mean\;}_{SE}=\;2.28$) in contrast with the other subsamples (${Mean\;}_{SBO}=\;2.33;\;{Mean\;}_{MSME}=\;2.68)$. In summary, smaller businesses are less aware of CC strategies than larger ones. To assess the consistency of these patterns, we analyzed the distribution across categories using a Pearson chi-square ($X^{2}$) homogeneity test. The results indicate statistically significant heterogeneous behavior in the samples regarding their awareness levels on various climate-related issues: (a) Global warming ($X^{2}=33.08;p=0.00)$; (b) Climate change ($X^{2}=25.10;p=\;0.00)$; (c) Greenhouse gas mitigation ($X^{2}=43.72;p=\;0.00)$; (d) Adaptation to climate change impacts ($X^{2}=31.17;p=\;0.00)$; (e) Offsetting of greenhouse gas emissions ($X^{2}=43.96;p=\;0.00)$; (f) Carbon neutrality ($X^{2}=32.49;p=\;0.00)$; and (g) Decarbonization ($X^{2}=28.58;p=\;0.00)$. These findings highlight a significant divergence in the business community’s awareness of climate-related issues. They also align with recent empirical research in developing regions [57,58], which has pointed to low levels of climate change awareness among ventures—limiting the successful implementation of strategies to address its effects. Moreover, the results underscore the need for tailored learning initiatives to raise awareness of environmental challenges, fostering engagement with sustainability practices.

### Risk perception: Who is most affected by climate change impact

Afterward, we examined the risk perception of each type of venture regarding CC. Respondents assessed potential impact pathways, distinguishing between internal risks (i.e., the initial and most prominent CC effects on the venture itself) and external risks (i.e., the initial and most prominent CC effects on business partners such as suppliers or customers). Table 3 presents the identified risk perception and the corresponding Perceived Impact Probability (PIP). Specifically, most of the sample considers an internal impact pathway as the most likely (PIP=0.54), followed by an external path (PIP=0.32). Additionally, a smaller proportion of respondents believe that CC will not affect anyone (PIP=0.09) or they lack enough information to perceive a risk (PIP=0.01).

**Table 3:** Climate Change Risk Perception.

Risk Perception Pathways	All Sample	SE	SBO	MSMEs
**Internal**	0.54	0.50	0.50	0.59
**External**	0.32	0.35	0.36	0.25
**None will be affected**	0.09	0.11	0.09	0.07
**No response**	0.05	0.03	0.04	0.07
**Not know**	0.01	0.01	0.02	0.01

Note. SBO = Small Business Owners; SE = Self-Employed; MSMEs = Micro, Small, and Medium-sized Enterprises.

Table 3 presents the results according to each subsample. The MSMEs subsample perceived an internal risk as most likely (${PIP}_{MSMEs}=0.59)$, in contrast to the other subsamples (${PIP}_{SBO}=0.50;\;{PIP}_{SE}=0.50)$. Besides, the SBO subsample perceived an external risk path as most likely (${PIP}_{SBO}=0.36)$, compared to other subsamples ${PIP}_{MSMEs}=0.25;\;{PIP}_{SE}=0.35)$. More so, we observe that the MSMEs $\left({PIP}_{MSMEs}=0.14\right)$ and SE $\left({PIP}_{SE}=0.14\right)$ subsamples showed the highest perception of no-risk compared to the other subsample $\left({PIP}_{SBO}=0.13\right)$. Lastly, the SBO subsample showed the highest response indicating a lack of awareness of risks $\left({PIP}_{SBO}=0.02\right)$ compared to the other subsamples $\left({PIP}_{MSMEs}=0.01;\;{PIP}_{SE}=0.01\right)$. As such, results suggest that MSMEs have varied perceptions regarding who will be most affected by CC. However, the prevalence of responses that prioritize internal stakeholders indicates a trend toward considering CC impacts within the organization’s direct sphere of influence.

To assess behavioral patterns among firms, we examined the distribution using a Pearson chi-square ($X^{2}$) homogeneity test. The results indicate statistically significant heterogeneity in the samples regarding their risk perception ($X^{2}=30.41;p=0.00)$. This finding suggests that ventures perceive climate risks differently, primarily influenced by their internal characteristics. Given their size, ventures must make strategic choices regarding key business functions (e.g., site location, capital allocation, labor management, and production patterns, among others), which shape their organizational exposure and vulnerability to climate-related pressures. The observed heterogeneity reinforces the importance of evaluating risks based on the resources and capabilities available to each venture while also considering potential variations in the physical, ecological, and socioeconomic systems in which they operate [59,60].

### Assessing strategic awareness: Climate change management

Afterward, we explored strategic awareness regarding CC management. Respondents were asked to describe their knowledge of strategies at both the corporate practice and operational process levels aimed at addressing the challenges their ventures face related to CC. Panel A of Table 4 reveals that most MSMEs demonstrate higher awareness of strategies at the corporate practice level compared to those aimed at operational processes. Specifically, 54.63 percent of the MSMEs identified corporate practice level strategies. Additionally, the SBO subsample showed the highest awareness regarding corporate practices level strategies (63.27 percent), while the MSMEs subsample exhibited the highest awareness at the operational processes level (27.04 percent). Moreover, we found that the SE subsample displayed the lowest awareness (11.02 percent) about strategies related to climate change compared to the other subsamples. These findings, supported by the Pearson chi-square ($X^{2}$) test of homogeneity ($X^{2}=32.49;p=\;0.00)$, reveal statistically significant heterogeneity across firms, regardless of economic sector. These patterns resonate with previous empirical findings [61], which posit that most organizations exhibit a unique combination of strategies at the corporate practice level (i.e., including carbon compensation, reduction, and independence) toward shaping their long-term carbon generation. This variation underscores the importance of tailored strategies depending on the organizational characteristics, size, and economic sector that may influence the organizations’ capacity to adopt and implement effective climate-related initiatives [62].

**Table 4:** Strategic Awareness.

Panel A. All sample (N = 1453)				
Strategic Awareness	SE	SBO	MSMEs	Grand Total
**At the level of corporate practices**	55.91	63.27	54.63	58.71
**At the level of operational processes**	24.80	20.83	27.04	23.88
**No response**	8.27	8.02	14.70	10.60
**Not aware**	11.02	7.87	3.63	6.81
**Total**	100.00	100.00	100.00	100.00
**Panel B. Primary sector (N = 89)**				
**Strategic Awareness**	**SE**	**SBO**	**MSMEs**	**Grand Total**
**At the level of corporate practices**	50.00	70.00	60.78	62.92
**At the level of operational processes**	25.00	16.67	13.73	15.73
**No response**	0.00	6.67	23.53	15.73
**Not aware**	25.00	6.67	1.96	5.62
**Total**	100.00	100.00	100.00	100.00
**Panel C. Secondary sector (N = 430)**				
**Strategic Awareness**	**SE**	**SBO**	**MSMEs**	**Grand Total**
**At the level of corporate practices**	66.67	67.65	58.93	63.02
**At the level of operational processes**	11.11	15.29	27.23	21.16
**No response**	11.11	8.82	10.71	10.00
**Not aware**	11.11	8.24	3.13	5.81
**Total**	100.00	100.00	100.00	100.00
**Panel D. Tertiary sector (N = 934)**				
**Strategic Awareness**	**SE**	**SBO**	**MSMEs**	**Grand Total**
**At the level of corporate practices**	54.29	61.16	50.00	56.32
**At the level of operational processes**	27.14	23.21	29.35	25.91
**No response**	8.10	7.81	16.30	10.39
**Not aware**	10.48	7.81	4.35	7.39
**Total**	100.00	100.00	100.00	100.00

Note. Values in percentages. SBO = Small Business Owners; SE = Self-Employed; MSMEs = Micro, Small, and Medium-sized Enterprises.

Furthermore, we examined strategic awareness at the economic sector level. Panel B of Table 4 reveals that ventures within the primary economic sector exhibit a higher strategic awareness at the corporate practice level compared to the operational process level. Indeed, a significant proportion of ventures in the primary sector of the economy indicated awareness of corporate practice strategies (62.92 percent), while a smaller proportion mentioned operational process strategies (15.73 percent). Notably, 23.53 percent of MSMEs in the primary sector did not respond, possibly indicating uncertainty or a lack of awareness regarding climate change strategies. Similarly, a substantial portion of SE ventures (25.00 percent) expressed lack of awareness of any strategy. In contrast, when examining the secondary economic sector, Panel C of Table 4 shows that MSMEs and SBO exhibit greater awareness of strategies at the corporate practice level (MSMEs= 58.93 percent; SBO=67.65 percent) compared to operational processes (MSMEs= 27.23 percent; SBO=15.29 percent). SE ventures in this sector demonstrated a higher awareness of strategies at the corporate practices level (66.67 percent), with a notable percentage (11.11 percent) indicating no response or lack of awareness. Finally, Panel D of Table 4 shows that SE and SBO in the tertiary economic sector exhibit higher awareness of strategies at the corporate practices level (SE = 54.29 percent; SBO = 61.16 percent) compared to MSMEs (50.00 percent). However, MSMEs in the tertiary sector demonstrated greater awareness of operational process strategies (29.35 percent) than SE (27.14 percent) and SBO (SBO = 23.21 percent).

The above findings, supported by the Pearson chi-square ($X^{2}$) test of homogeneity, indicate significant heterogeneity across economic sectors ($Primary:\;X^{2}=80.49;Secondary:\;X^{2}=54.32;\;Tertiary:\;X^{2}=54.32;\;p=\;0.00)$, reinforcing the notion that ventures across different sectors exhibit varied levels of strategic CC awareness. According to previous empirical studies [63], the impact and strategic response to CC vary across the business community. Indeed, based on our analysis, MSMEs exhibit heterogeneous strategic approaches to cope with the increased pressures on the operational viability of businesses, which may be constrained by the resources available within the organization [62]. These sector-specific variations emphasize the need for tailored CC strategies and educational efforts that address the unique challenges and opportunities faced by firms from different sectors.

### Carbon neutrality: Strategic timing and antecedents

Subsequently, we explored the period upon which MSMEs expect to achieve carbon neutrality. Table 5 details the strategic timing for each type of MSME. Panel A shows that a significant proportion of respondents believed they could achieve carbon-neutral emissions within five years (33.72 percent), while 42.53 percent expected to reach this goal within six to twenty years. However, a notable share of respondents expressed either a lack of interest in carbon neutrality (15.62 percent), skepticism about its feasibility (13.08 percent), or insufficient information to estimate its timing (10.67 percent). Furthermore, the Pearson chi-square ($X^{2}$) test of homogeneity ($X^{2}=42.01;p=\;0.00)$ revealed statistically significant heterogeneity in strategic timing across SEs, SBOs, and MSMEs, indicating that these groups follow varied timelines for achieving carbon neutrality. This suggests a lack of a unified societal objective for reaching this goal. Moreover, these results align with recent discussions on climate neutrality, that stress the absence of standardized global guidelines for establishing net-zero strategies [64,65]). This lack of clear directives has led to challenges in setting socially accepted carbon targets and designing appropriate strategies, with the approach to carbon neutrality largely influenced by company size.

**Table 5:** Carbon Neutral: Strategic Timing.

Panel A. All sample (N = 1,453)				
Strategic Timing	SE	SBO	MSMEs	Grand Total
**Period less than or equal to 5 years**	27.56	32.10	38.48	33.72
**From 6 to 10 years**	11.81	19.60	21.60	19.00
**11 to 20 years**	8.27	4.32	4.72	5.16
**More than 20 years**	3.15	3.24	2.00	2.75
**Not in the company’s interest**	19.69	15.43	13.97	15.62
**Do not see it feasible**	18.11	14.81	8.71	13.08
**Don’t know**	11.42	10.49	10.53	10.67
**Grand Total**	100	100	100	100
**Panel B. Primary sector (N = 89)**				
**Strategic Timing**	**SE**	**SBO**	**MSMEs**	**Grand Total**
**Period less than or equal to 5 years**	50.00	40.00	56.86	50.56
**From 6 to 10 years**	25.00	30.00	9.80	17.98
**11 to 20 years**	0.00	3.33	7.84	5.62
**More than 20 years**	0.00	3.33	1.96	2.25
**Not in the company’s interest**	0.00	10.00	3.92	5.62
**Do not see it feasible**	25.00	6.67	9.80	10.11
**Don’t know**	0.00	6.67	9.80	7.87
**Grand Total**	100	100	100	100
**Panel C. Secondary sector (N = 430)**				
**Strategic Timing**	**SE**	**SBO**	**MSMEs**	**Grand Total**
**Period less than or equal to 5 years**	22.22	29.41	29.91	29.07
**From 6 to 10 years**	8.33	18.82	25.89	21.63
**11 to 20 years**	11.11	4.12	4.46	4.88
**More than 20 years**	8.33	2.94	3.57	3.72
**Not in the company’s interest**	22.22	15.29	14.29	15.35
**Do not see it feasible**	11.11	18.24	12.05	14.42
**Don’t know**	16.67	11.18	9.82	10.93
**Grand Total**	100	100	100	100
**Panel D. Tertiary sector (N = 934)**				
**Strategic Timing**	**SE**	**SBO**	**MSMEs**	**Grand Total**
**Period less than or equal to 5 years**	27.62	32.59	42.03	34.26
**From 6 to 10 years**	11.90	19.20	20.29	17.88
**11 to 20 years**	8.10	4.46	4.35	5.25
**More than 20 years**	2.38	3.35	0.72	2.36
**Not in the company’s interest**	20.00	15.85	15.58	16.70
**Do not see it feasible**	19.05	14.06	5.80	12.74
**Don’t know**	10.95	10.49	11.23	10.81
**Grand Total**	100	100	100	100

Note. Values in percentages. SBO = Small Business Owners; SE = Self-Employed; MSMEs = Micro, Small, and Medium-sized Enterprises.

In turn, panels B, C, and D in Table 5 present the strategic timing by economic sector. We observe that most firms in the primary sector perceive achieving carbon neutrality in a period lower than 10 years (${Mean\;}_{Primary\;Sector}=0.71)$, a proportion that is higher than others economic sectors (${Mean\;}_{Secondary\;Sector}=0.45;\;{Mean\;}_{Tertiary\;Sector}=0.51)$. This suggests that MSMEs in the primary sector view carbon neutrality as critical for survival. The Pearson chi-square ($X^{2}$) test of homogeneity indicates homogeneity among business ventures within the primary sector ($X^{2}=11.90;p=\;0.454)$. This suggests that businesses within this sector may share a common strategic approach, possibly viewing carbon neutrality as a critical component achievable in the short term. More so, we observe that a higher proportion of MSMEs from the secondary sector (${Mean\;}_{Secondary\;Sector}=0.12)$ perceive achieving carbon neutrality in a period higher than 10 years but lower than twenty years. This proportion is higher than others economic sectors (${Mean\;}_{Primary\;Sector}=0.05;\;{Mean\;}_{Tertiary\;Sector}=0.08)$. This result could imply that MSMEs from the secondary sector may plan and invest with a long-term perspective to achieve significant changes in their production processes. This perspective is further supported by the Pearson chi-square ($X^{2}$) test of homogeneity, which indicates homogeneity among ventures within this sector ($X^{2}=17.06;p=\;0.148)$, thus, suggesting a sector-wide acknowledgment of the inherent complexities in attaining carbon neutrality in the short term. This alignment within the secondary sector could point to common strategic planning practices aimed at balancing operational feasibility with long-term sustainability goals.

Furthermore, we remark that a significant proportion of firms in the secondary and tertiary sectors believe that carbon neutrality is either not of interest to them or not feasible to achieve (${Mean\;}_{Secondary\;Sector}=0.31;\;{Mean\;}_{Tertiary\;Sector}=0.30)$. This proportion is larger than in the primary sector (${Mean\;}_{Primary\;Sector}=0.18)$, suggesting that most MSMEs do not see a direct benefit in pursuing non-financial strategies like carbon neutrality. More so, results show that MSMEs from the secondary sector exhibit a lower awareness regarding strategic timing for achieving carbon neutrality (${Mean\;}_{Secondary\;Sector}=0.13)$, in contrast to MSMEs from other economic sectors (${Mean\;}_{Primary\;Sector}=0.05;\;{Mean\;}_{Tertiary\;Sector}=0.11)$. This overall trend may suggest that ventures across different economic sectors lack specific knowledge about strategic planning on sustainability matters. To further explore our initial findings, we conducted a Logistic regression analysis focusing on two interrelated outcomes: (a) the perception that carbon neutrality is achievable within a five-year timeframe (Model ‘a’), and (b) the view that carbon neutrality is either unattainable or not a current strategic priority (Model ‘b’). S1 and S2 Tables present the univariable and bivariate analyses of the variables, indicating no evidence of multicollinearity and suggesting independence between observations. These findings support the core assumptions of the model.

Table 6 presents the odds ratios (OR) and marginal effects (ME) derived from this analysis. The results in Model ‘a’ highlight key factors influencing short-term carbon neutrality perceptions. Notably, endorsing gender-balanced leadership did not have a statistically significant effect on perceiving carbon neutrality as achievable within five years (OR= 1.01; ME= 0.00; p = 0.96), suggesting that other factors might play a more decisive role in shaping organizational attention toward this strategy. Additionally, capital barriers exhibited a positive but non-significant effect (OR=1.20; ME= 0.03; p = 0.15), while technology barriers showed a negative yet non-significant effect (OR= 0.88; ME= −0.03; p = 0.34). These findings suggest that ventures’ decisions to engage in long-term climate-related strategies may be driven more by managerial choices than by the availability of specific resources.

**Table 6:** Carbon Neutrality Strategic Timing.

Panel 1.												
Model ‘a’	Estimates						Delta-Method					
	OR	SD	P-value		95% CI		ME	SD	P-value		95% CI	
**Gender-balanced leadership**	1.01	0.13	0.96		0.79	1.29	0.00	0.03	0.96		-0.05	0.06
**Capital barriers**	1.20	0.15	0.15		0.94	1.53	0.04	0.03	0.15		-0.01	0.09
**Technology barriers**	0.88	0.12	0.34		0.67	1.14	-0.03	0.03	0.34		-0.09	0.03
**Government support**	2.87	2.92	0.30		0.39	21.07	0.23	0.22	0.30		-0.21	0.67
**Stakeholder support**	0.33	0.38	0.34		0.03	3.18	-0.25	0.26	0.34		-0.75	0.25
**Customer pressures**	1.25	0.20	0.17		0.91	1.70	0.05	0.04	0.17		-0.02	0.12
**Investor pressures**	1.50	0.24	0.01	**	1.10	2.06	0.09	0.04	0.01	**	0.02	0.16
**Awareness of carbon markets**	1.33	0.17	0.03	**	1.03	1.72	0.06	0.03	0.03	**	0.01	0.12
**Carbon-related practices**	1.22	0.22	0.27		0.86	1.72	0.04	0.04	0.27		-0.03	0.12
**Carbon programs**	1.49	0.20	0.00	***	1.14	1.94	0.09	0.03	0.00	***	0.03	0.15
**Economic Sector**												
**Secondary Sector**	0.40	0.10	0.00	***	0.25	0.65	-0.21	0.06	0.00	***	-0.32	-0.09
**Tertiary Sector**	0.59	0.14	0.02	**	0.37	0.93	-0.13	0.06	0.03	**	-0.24	-0.01
**Business Community**												
**Small business owner**	1.20	0.21	0.28		0.86	1.69	0.04	0.04	0.27		-0.03	0.11
**MSMEs**	1.55	0.29	0.02	**	1.08	2.23	0.10	0.04	0.01	**	0.02	0.17
**Constant**	0.35	0.10	0.00	***	0.20	0.63						
**Observations**	1453.00											
**Pseudo R square**	0.04											
**Panel 2.**												
**Model ‘b’**	**Estimates**						**Delta-Method**					
	**OR**	**SD**	**P-value**		**95% CI**		**ME**	**SD**	**P-value**		**95% CI**	
**Gender-balanced leadership**	0.70	0.09	0.01	**	0.54	0.90	-0.08	0.03	0.01	**	-0.14	-0.03
**Capital barriers**	0.66	0.08	0.00	***	0.52	0.83	-0.10	0.03	0.00	***	-0.15	-0.04
**Technology barriers**	0.91	0.12	0.47		0.70	1.18	-0.02	0.03	0.47		-0.08	0.04
**Government support**	1.12	1.14	0.92		0.15	8.30	0.03	0.24	0.92		-0.45	0.50
**Stakeholder support**	1.56	1.39	0.61		0.27	8.91	0.11	0.21	0.61		-0.31	0.52
**Customer pressures**	0.65	0.10	0.00	***	0.49	0.88	-0.10	0.04	0.00	***	-0.17	-0.03
**Investor pressures**	0.65	0.10	0.01	**	0.49	0.88	-0.10	0.04	0.01	**	-0.17	-0.03
**Awareness of carbon markets**	0.67	0.09	0.00	***	0.51	0.87	-0.10	0.03	0.00	***	-0.16	-0.03
**Carbon-related practices**	0.49	0.10	0.00	***	0.33	0.74	-0.17	0.05	0.00	***	-0.26	-0.07
**Carbon programs**	0.64	0.09	0.00	***	0.48	0.85	-0.10	0.03	0.00	***	-0.17	-0.04
**Economic Sector**												
**Secondary Sector**	1.99	0.57	0.02	**	1.13	3.48	0.15	0.06	0.01	**	0.04	0.27
**Tertiary Sector**	1.65	0.46	0.07	*	0.96	2.84	0.11	0.06	0.05	*	0.00	0.22
**Business Community**												
**Small business owner**	0.83	0.13	0.25		0.60	1.14	-0.05	0.04	0.25		-0.12	0.03
**MSMEs**	0.67	0.12	0.02	**	0.47	0.94	-0.10	0.04	0.02	**	-0.18	-0.01
**Constant**	1.64	0.52	0.12		0.89	3.05						
**Observations**	1453.00											
**Pseudo R square**	0.07											

Note. The table presents estimates and marginal effects from a logit model assessing the relevance of firms’ efforts to achieve carbon neutrality. Model ‘a’´ examines whether the firm perceives achieving carbon neutrality within less than five years as feasible. Model ‘b’ examines whether the firm considers carbon neutrality infeasible or does not include it in its strategic plan. OR = Odds Ratios; SD = Robust Standard Errors; CI = Confidence Intervals. ***p-value < 0.001; ** p-value < 0.05; * p-value < 0.10.

Furthermore, ventures perceiving greater investor pressure to pursue carbon neutrality were significantly more likely to consider this goal feasible (OR= 1.50; ME= 0.09; p = 0.01). In contrast, the perceived support from the government (OR= 2.87; ME= 0.23; p = 0.30) and customer pressures (OR= 1.25; ME= 0.05; p = 0.17) had positive but non-significant effects. Similarly, perceived support from stakeholders, such as employees, had a negative but non-significant effect (OR= 0.33; ME= −0.25; p = 0.34). Moreover, ventures with greater awareness of domestic carbon markets (OR=1.33; ME= 0.06; p = 0.03) and those already implementing carbon management initiatives within their operations (OR=1.49; ME= 0.09; p = 0.00) were significantly more likely to perceive carbon neutrality as achievable in the short term. Conversely, firms in the secondary (OR=0.40; ME= −0.21; p = 0.00) and tertiary (OR=0.59; ME = −0.13; p = 0.03) sectors exhibited a lower likelihood of adopting short-term carbon neutrality goals, possibly reflecting sector-specific challenges in aligning sustainability initiatives with existing operational structures. Finally, MSMEs demonstrated a higher likelihood of perceiving carbon neutrality as feasible within five years (OR=1.55; ME = 0.10; p = 0.01), suggesting a proactive stance among these ventures in adapting to sustainability objectives.

Model ‘b’ highlights several factors that reduce the likelihood of perceiving carbon neutrality as infeasible or not endorsing this strategic goal. At the governance level, firms with gender-balanced leadership teams are less likely to dismiss carbon neutrality as an unattainable objective (OD=0.70; ME= −0.08; p = 0.01). This aligns with existing empirical research [66], which identifies gender diversity as a significant predictor of organizational environmental initiatives. In the context of MSMEs, this suggests that a gender-balanced governance structure may contribute to reducing carbon emissions while simultaneously supporting the organization’s long-term profitability and resilience. Moreover, the results indicate that perceived capital (OD=0.66; ME= −0.10; p = 0.00and technology (OD=0.91; ME= −0.02; p = 0.47) barriers reduce the likelihood of considering carbon neutrality as infeasible or excluding it from strategic planning. This finding suggests that MSMEs may perceive environmental value creation as a potential driver of growth. It is plausible that having sufficient internal resources increases the likelihood of integrating carbon neutrality into strategic planning. Further research is needed to determine the specific resources required to facilitate this integration. Additionally, ventures perceiving pressures from customers (OD=0.65; ME= −0.10; p = 0.00) and investors (OD=0.67; ME= −0.10; p = 0.01) are less likely to disregard climate-related strategies. In contrast, perceived support from the government (OD=1.12; ME= 0.03; p = 0.92) and stakeholders, such as employees, (OD=1.56; ME= 0.11; p = 0.61) exhibit a positive but non-statistically significant effect. These findings suggest that perceived normative discourses might play a critical role in shaping the strategic decisions of MSMEs regarding climate action.

Finally, awareness of national carbon markets reduces the likelihood of perceiving carbon neutrality as unfeasible (OD=0.67; ME= −0.10; p = 0.00), suggesting that familiarity with these markets positively influences strategic alignment with sustainability goals. At the corporate level, ventures actively engaged in carbon-related practices (OD=0.49; ME = −0.17; p = 0.00) or implementing carbon programs (OD=0.64; ME = −0.10; p = 0.00) are similarly less likely to view carbon neutrality as unattainable, highlighting a potential learning effect associated with sustainability initiatives. Conversely, ventures in the secondary (OD=1.99; ME= 0.15; p = 0.01) and tertiary (OD=1.65;  ME= 0.11; p = 0.05) sectors are more inclined to perceive carbon neutrality as less feasible, potentially due to sector-specific constraints. Lastly, MSMEs demonstrate a lower likelihood of dismissing carbon neutrality as a strategic option (OD=0.67; ME = −0.10; p = 0.02), suggesting that they may view sustainability commitments as essential for long-term survival.

### Carbon neutrality: Strategic awareness

At last, we explored MSMEs’ strategic awareness regarding carbon neutrality (please refer to Table 7 for an overview). Specifically, MSMEs aim to implement carbon neutrality strategies at either the corporate practice or operational process level to achieve their strategic timing. Panel A outlines the overall trend observed within the sample. This trend reveals most respondents (${Mean\;}_{All}=0.41)$ intend to implement strategies at the operational process level, while a smaller proportion anticipates implementing strategies at the corporate practice level (${Mean\;}_{All}=0.14).$ Remarkably, a significant proportion of participants did not respond (${Mean\;}_{All}=0.45)$, indicating a potential lack of understanding on how to effectively pursue carbon neutrality. To further explore this result, we conducted a Pearson chi-square ($X^{2}$) test of homogeneity which suggests that the sample behaves in an heterogenous way ($X^{2}=115.94;p=\;0.00)$ further confirming the heterogeneity on strategic planning within the business community. These findings provide further evidence of the absence of standardized global guidelines for establishing net-zero strategies [64]. This lack of clear pathways for selecting carbon neutrality strategies hinders executives from effectively driving environmentally positive organizations. While some conceptual studies have attempted to address this gap, further research is needed to develop clearer and more actionable guidelines for the business community.

**Table 7:** Carbon Neutral: Strategies.

Panel A. All sample (N = 1,453)				
Strategic Awareness	SE	SBO	MSMEs	Grand Total
**At the level of operational processes**	48.28	41.67	33.46	42.74
**At the level of corporate practices**	12.16	14.35	14.96	13.63
**No response**	39.56	43.98	51.57	43.63
**Total**	100	100	100	100
**Panel B. Primary sector (N = 89)**				
**Strategic Awareness**	**SE**	**SBO**	**MSMEs**	**Grand Total**
**At the level of operational processes**	52.94	46.67	62.50	51.69
**At the level of corporate practices**	13.73	26.67	12.50	17.98
**No response**	33.33	26.67	25.00	30.34
**Total**	100	100	100	100
**Panel C. Secondary sector (N = 430)**				
**Strategic Awareness**	**SE**	**SBO**	**MSMEs**	**Grand Total**
**At the level of operational processes**	46.43	37.65	38.89	42.33
**At the level of corporate practices**	12.50	14.12	8.33	12.79
**No response**	41.07	48.24	52.78	44.88
**Total**	100	100	100	100
**Panel D. Tertiary sector (N = 934)**				
**Strategic Awareness**	**SE**	**SBO**	**MSMEs**	**Grand Total**
**At the level of operational processes**	48.91	42.86	31.43	42.08
**At the level of corporate practices**	11.59	13.62	16.19	13.60
**No response**	39.49	43.53	52.38	44.33
**Total**	100	100	100	100

Note. Values in percentages. SBO = Small Business Owners; SE = Self-Employed; MSMEs = Micro, Small, and Medium-sized Enterprises.

Likewise, panels B, C, and D in Table 7 describe the strategic awareness among MSMEs by economic sector. It is observed that ventures in the primary sector (${Mean\;}_{Primary\;Sector}=0.54$are more inclined to engage in strategies at the operational process level compared to those from other economic sectors (${Mean\;}_{Secondary\;Sector}=0.41;\;{Mean\;}_{Tertiary\;Sector}=0.41)$. This trend may suggest that certain sectors find it easier to integrate operational-level strategies into their approach to achieving carbon neutrality, due to their operational nature. Besides, we observe that ventures in the primary sector (${Mean\;}_{Primary\;Sector}=0.18)$ and tertiary sector (${Mean\;}_{Tertiary\;Sector}=0.18$are more likely to engage in corporate practices level compared to the secondary sector (${Mean\;}_{Secondary\;Sector}=0.14)$. This observation could imply that ventures from the secondary sector may need to engage in long-term planning to balance financial and non-financial strategies effectively.

Finally, we found that ventures from the secondary (${Mean\;}_{Secondary\;Sector}=0.47)$ and tertiary sector (${Mean\;}_{Tertiary\;Sector}=0.45)$ had the highest proportion of no response about carbon neutrality strategies compared to the primary sector ${(Mean\;}_{Primary\;Sector}=0.28)$. This may suggest that some economic sectors may lack knowledge about the concept of “Carbon Neutrality” and how to strategize and potentially implement it effectively. These findings are further validated by the Pearson chi-square ${(X}^{2}$) test of homogeneity, which demonstrates statistically significant heterogeneity in strategic awareness across sectors. Specifically, firms in the primary ($X^{2}=54.32;p=\;0.00)$, secondary ($X^{2}=57.29;p=\;0.00)$, and tertiary ($X^{2}=80.49;p=\;0.00$sectors exhibit distinct behaviors in their approach to strategic awareness. This sector-based heterogeneity suggests that sector-specific characteristics influence how organizations prioritize and integrate sustainability and carbon neutrality within their strategic frameworks. These findings align with previous research [64,65], highlighting the need for tailored approaches that consider sectoral differences.

## Discussion

Given the relevance of guiding MSMEs toward a greener economy, we aimed to answer three interrelated research questions: a) To what extent are MSMEs aware of CC and decarbonization? (b) How do MSMEs develop strategies to address CC and achieve carbon neutrality? (c) What factors shape MSMEs’ attention to CC and decarbonization? Table 8 presents a comparative summary of our main findings alongside those reported in prior empirical studies. It highlights areas of consistency, divergence, and potential contributions to ongoing scholarly debates. Our findings indicate that while MSMEs are generally familiar with broad concepts such as “climate change” and “global warming,” their understanding of more specific terms like “carbon neutrality” and “decarbonization” remains limited. Additionally, we observed significant disparities in climate-related knowledge across the business community, which may impact strategic decision-making. Furthermore, MSMEs perceive higher internal perceived institutional pressures (PIP) than external PIP, suggesting that shifts in production and operational processes may influence how these organizations create value. Across economic sectors, most ventures showed greater awareness of strategies at the corporate practice level than at the operational process level.

**Table 8:** Comparison of Results with Prior Empirical Research.

Extant Literature	Key insights
As suggested by [32], scientific knowledge often diverges from public understanding, highlighting the need for targeted educational programs specifically designed for the MSME population.	MSMEs only possess a basic understanding of CC, while more specialized or advanced aspects of the topic remain largely unfamiliar to them (i.e., in line with previous literature).
As noted by [33], firms with limited human and financial resources tend to be more vulnerable to climate risks, which leads them to perceive a greater internal impact.	Ventures perceive climate risk in diverse ways, influenced by internal characteristics such as firm size and stage of development (i.e., supports previous literature).
As previous research suggests [26], organizational objectives may often be misaligned with climate-related outcomes due to existing knowledge gaps concerning their environmental impacts.	Ventures across economic sectors exhibit varying levels of strategic climate change (CC) awareness, with firms in the primary sector demonstrating a higher awareness of corporate sustainability strategies (i.e., as stated in previous research).
Previous studies (e.g., [37]; [35]) suggest that external pressures often serve as catalysts for the adoption of proactive sustainability strategies.	Ventures tend to adopt more proactive carbon neutrality strategies when they perceive strong external pressures, such as those exerted by investors (i.e., as shown in previous studies).
Empirical evidence [25] suggests that customer pressure serves as a driver of proactive environmental practices.	Our results indicate a positive, albeit statistically non-significant, effect of customer pressure on firms’ short-term strategic timing for achieving carbon neutrality. This outcome may be explained by the influence of other stakeholders, such as investors, who may play a more prominent role in shaping strategic environmental decisions within this contextual setting (i.e., in contrast to previous literature).
Evidence suggests [34] that internal environmental policies and practices are related to environmental performance	Our regression analysis provides mixed positive evidence on the influence of internal carbon-related practices and programs in shaping short-term strategic timing for achieving carbon neutrality (i.e., to an extent, similar to previous research).

Our analysis also revealed substantial heterogeneity in strategic timing for achieving carbon neutrality, though we observed notable homogeneity in timing within the primary and secondary sectors. Investor pressures, awareness of carbon markets, and participation in carbon programs were linked to a greater likelihood of firms perceiving carbon neutrality as achievable within five years. Conversely, MSMEs with gender-balanced governance, capital constraints, and external pressures from customers and investors—along with engagement in carbon-related initiatives—were less likely to dismiss carbon neutrality or exclude it from their strategic planning. In addition, our findings highlight significant variation in carbon neutrality strategies among MSMEs, pointing to a lack of clear guidelines on how to effectively implement such strategies. Based on these insights, we now present the theoretical implications, and public policy and practical recommendations derived from this project.

### Theoretical implications

Our study contributes to the ABV approach by illustrating how MSMEs’ responses to climate CC are shaped by their contextual environment, particularly ecological pressures and normative discourses. ABV posits that firms allocate their attention to issues they perceive as most pressing, which, in the case of MSMEs, is influenced by sectoral characteristics, resource availability, and the salience of sustainability-related pressures. Our study reveals that while MSMEs generally recognize broad climate-related concepts, their understanding of more technical terms like carbon neutrality and decarbonization is limited. This suggests that MSMEs’ strategic attention is unevenly distributed across the business community, with some firms prioritizing CC adaptation while others focus on short-term financial concerns. The observed heterogeneity aligns with ABV’s assertion that organizational attention is constrained by cognitive limits and contextual drivers, influencing which sustainability strategies are perceived as viable.

A key implication of our study is the role of sector-based heterogeneity in shaping firms’ attention to sustainability. While some sectors demonstrate greater alignment with sustainability practices, others exhibit weaker engagement, likely due to differences in operational needs, regulatory pressures, and market incentives. This finding highlights the need for more targeted theoretical frameworks that account for sector-specific variations in how organizations interpret and respond to climate-related issues. Future research should explore how different industry dynamics influence firms’ prioritization of CC and sustainability, thereby expanding ABV’s applicability to sectoral decision-making. Our results also highlight the impact of internal organizational factors on sustainability attention. MSMEs experiencing stronger internal institutional pressures—such as shifts in production processes—appear more inclined to incorporate sustainability into their strategic priorities. This suggests that firms’ internal structures, governance models, and decision-making processes play a crucial role in determining how sustainability is framed within their strategic agenda. Specifically, our findings support recent empirical work that links gender-balanced governance structures with greater environmental engagement. From an ABV perspective, this implies that governance composition may act as a filter through which sustainability pressures are processed and prioritized. Future studies could extend this analysis by investigating how internal leadership structures shape firms’ cognitive processing of ecological pressures.

Additionally, our findings underscore the absence of standardized global guidelines for establishing net-zero strategies, which hinders MSMEs from effectively translating environmental awareness into actionable sustainability practices. This lack of clear directives reinforces ABV’s premise that firms selectively process external pressures, focusing on those that offer clearer strategic pathways. Without well-defined institutional frameworks guiding carbon neutrality efforts, MSMEs may struggle to allocate attention to sustainability in a meaningful way. Theoretical extensions of ABV could explore how the presence (or absence) of institutional clarity affects firms’ ability to navigate complex sustainability landscapes. Finally, while our study primarily employs a macro-level perspective of ABV, we acknowledge the critical role of micro-level decision-making dynamics. Prior research suggests that managerial cognition, risk perception, and leadership orientations significantly influence which issues gain organizational attention. Our findings suggest that MSMEs’ engagement with CC is not only a function of external pressures but also of internal managerial beliefs and resource availability. Future research could extend ABV by examining how micro-level cognitive frames interact with macro-level pressures to shape strategic sustainability decisions. By integrating these insights, scholars could develop a more comprehensive understanding of how MSMEs navigate environmental uncertainty and embed sustainability within their long-term strategic planning.

### Public policy implications

Our findings highlight critical disparities in MSMEs’ awareness and strategic engagement with CC and decarbonization, underscoring the need for targeted policy interventions. Given the heterogeneous levels of knowledge and engagement across economic sectors, broad policy initiatives should be complemented by industry-specific measures that address distinct challenges faced by MSMEs. While MSMEs generally recognize broad CC-related concepts, their understanding of technical terms such as carbon neutrality and decarbonization remains limited. For example, we found particularly pronounced knowledge gaps in service-oriented industries. This knowledge gap impedes their ability to implement effective sustainability strategies. As such, public policies should prioritize tailored training and educational programs that reflect sectoral needs, providing industry-specific guidance on sustainability strategies. Outreach programs should translate complex CC concepts into actionable insights using accessible language, real-world case studies, and digital training modules. Additionally, governments should mandate sustainability literacy programs as part of business licensing and certification processes, ensuring that MSMEs integrate environmental considerations into their strategic planning from inception. Furthermore, public awareness campaigns should highlight successful MSMEs implementing CC practices, using case studies, awards, and certification programs to incentivize broader participation. Overall, given the sectoral differences in CC awareness and response strategies, policy frameworks should be adaptable to specific industry needs. For instance, MSMEs in agriculture and mining may require immediate climate adaptation strategies due to direct exposure to environmental risks, while manufacturing and service-based ventures may benefit more from structured long-term incentives for integrating sustainability into operations.

Our findings suggest that firms engaged in carbon-related initiatives are more likely to incorporate carbon neutrality into their strategic planning. However, many MSMEs lack the internal resources and expertise to develop comprehensive CC strategies. To bridge this gap, policymakers should foster inter-organizational collaboration through public-private partnerships, industry consortia, and government-led initiatives that connect MSMEs with larger firms, research institutions, and international sustainability programs. Sector-based collaborations, such as industry associations and chambers of commerce, should receive policy support to provide MSMEs with tailored assistance, including energy audits, operational efficiency upgrades, and sustainability-focused training programs. Institutionalizing these partnerships through formal agreements would ensure sustained engagement and long-term capacity-building efforts. In this same spirit, local governments could adopt a “street-level bureaucracy” approach, engaging MSMEs through advisory programs and accessible workshops to help businesses navigate CC regulations. Furthermore, the absence of standardized global guidelines for net-zero strategies creates uncertainty for MSMEs seeking to implement sustainability measures. Indeed, our findings reveal substantial variation in carbon neutrality strategies, further emphasizing the need for clear policy directives. Therefore, governments should develop sector-specific roadmaps outlining best practices, regulatory expectations, and achievable carbon reduction targets tailored to different industries. Regulatory clarity would help MSMEs navigate the complexities of sustainability transitions and align their strategies with broader national and international climate objectives.

Financial constraints also remain a significant barrier to MSMEs’ adoption of sustainability strategies. Our findings indicate that ventures with capital constraints are less likely to actively pursue carbon neutrality. To address this, policymakers should expand access to green financing mechanisms, including low-interest loans, grants, and subsidies for energy-efficient technologies. Additionally, tax incentives should be introduced for investments in renewable energy adoption, low-emission supply chain improvements, and carbon neutrality initiatives. Establishing national green credit lines tailored to MSMEs could provide financial support without jeopardizing operational stability. Linking financial incentives to local green certifications could further encourage participation by offering reputational advantages alongside financial benefits. Overall, ensuring long-term funding and consistent monitoring mechanisms could be crucial to supporting MSMEs in progressively developing their environmental capabilities without facing abrupt financial burdens.

### Practical implications

To translate climate awareness into actionable strategies, MSMEs require industry-specific guidance that aligns with their operational realities. Our study underscores the uneven distribution of climate-related knowledge across sectors, which requires practical solutions tailored to business size, sector, and available resources. Indeed, MSMEs across different sectors exhibit varied levels of strategic CC awareness, with many focusing on corporate-level sustainability initiatives rather than operational process improvements. To face this concern, business associations could develop toolkits outlining industry-specific steps for assessing carbon footprints and implementing sustainable practices. For example, agricultural MSMEs could receive guidance on climate-resilient farming techniques, while manufacturing firms could focus on energy efficiency and waste reduction strategies. Retail and service-based businesses, which often lack clear decarbonization roadmaps, could be provided with practical frameworks for integrating sustainability into supply chains and customer engagement strategies.

Our findings also indicate that firms engaged in carbon-related initiatives are more likely to perceive carbon neutrality as an achievable goal. MSMEs should explore opportunities in voluntary carbon markets, sustainability certification programs, and cooperative purchasing agreements for green technologies. Governments and industry associations should provide MSMEs with training on the financial benefits of carbon market participation, enabling them to capitalize on new revenue streams linked to carbon credits and emissions reductions. Even though many MSMEs perceive environmental value creation as a potential driver of growth, integrating CC strategies into core business functions remains a challenge. To address this, businesses should conduct environmental risk assessments to evaluate their exposure to climate-related disruptions. By incorporating climate considerations into site location decisions, capital allocation, labor management, and supply chain planning, MSMEs could proactively develop resilience against future sustainability challenges.

As we have shown, many MSMEs lack the internal expertise needed for effective environmental management. To overcome this challenge, industry associations and chambers of commerce should facilitate collective climate initiatives, such as cooperative sustainability programs, shared infrastructure for green technologies, and joint procurement of renewable energy solutions. Establishing knowledge-sharing networks among MSMEs could help smaller firms access sustainability resources at reduced costs while collectively enhancing their market competitiveness. Additionally, MSMEs should take advantage of available government programs, grants, and advisory services aimed at supporting sustainability transitions. By staying informed about regulatory changes and actively participating in policy discussions, businesses could better anticipate and adapt to evolving environmental requirements. Finally, forming partnerships with larger firms and research institutions could provide MSMEs with access to technical expertise, funding opportunities, and innovative sustainability solutions. Overall, by strengthening these practical support mechanisms, MSMEs could move beyond general climate awareness and adopt tangible, sector-specific strategies that contribute to long-term environmental sustainability and business resilience.

### Limitations

This project is not without limitations. A primary concern is the potential for response bias, as the data relies on self-reported perceptions and strategies from MSME C-level executives. This may lead to an overestimation or underestimation of their awareness and engagement with CC issues. Future research could incorporate qualitative approaches to explore the micro-foundations of CC-related decision-making. Complementary research designs may also help identify additional factors influencing MSMEs’ awareness and engagement with sustainability initiatives. Additionally, the study’s reliance on survey methods may not fully capture the nuanced understanding and complex mechanisms involved in how MSMEs address climate change. This approach could overlook the significant influence of external factors, such as government policies, market dynamics, and technological advancements, which are crucial in shaping MSMEs’ environmental awareness, behaviors, and strategies. Future research should adopt a more comprehensive approach that examines both internal and external drivers, offering deeper insights into how these factors collectively shape MSMEs’ responses to climate change.

Second, although the sample is representative of MSMEs in Colombia—a country within Latin America, a region often regarded as fertile ground for theory building and testing [47]—our findings are limited to contexts that share similar institutional environments and experience varying degrees of CC impact. This limitation affects the generalizability of the results, highlighting the need for further research in diverse settings to empirically validate our conclusions. Third, the study’s cross-sectional design presents another limitation, as it captures MSMEs’ perceptions and strategies at a single point in time. Consequently, this approach does not account for temporal changes or the evolution of attitudes and CC-related practices. To address this, future research should incorporate longitudinal studies to examine how MSMEs’ awareness and strategies develop over time.

Fourth, this study does not fully capture the complexity and diversity of CC impacts and responses. While it focuses on key themes such as awareness, risk perceptions, and strategic planning, other critical aspects—such as supply chain vulnerabilities, stakeholder engagement, and the socio-economic implications of CC strategies—may have been overlooked. Future research should explore these dimensions to provide a more comprehensive understanding of MSMEs’ engagement with CC-related challenges. Fifth, while this study aimed to identify general patterns within the business community regarding climate change awareness and related strategies, the use of anonymized data limited our ability to examine the decision-making processes through which firms select and implement specific climate change strategies and pursue carbon neutrality goals. Future research could address this gap by conducting case-based analyses of ventures successfully implementing carbon reduction initiatives and climate-related plans and programs. Such an approach could help identify commonalities and best practices across economic sectors, informing the development of more effective corporate sustainability programs. Additionally, this could facilitate the creation of novel frameworks to guide policy development in this area.

Sixth, while our empirical design identified key insights based on patterns and commonalities within the business community, it did not account for the financial, organizational, and operational resources and capabilities of each venture when endorsing specific CC-related strategies and goals. Future research could build on our findings by developing and testing hypotheses that focus on specific groups of MSMEs within the same economic sector, examining how venture-level characteristics either constrain or facilitate the strategic adoption of CC initiatives. Additionally, future studies could explore potential moderation and mediation effects influencing firms’ engagement in CC-related strategies. Finally, our research builds on existing theoretical insights into organizational awareness to shape our analysis. Given the various determinants influencing CC awareness and strategies, future research could develop new theories that account for the heterogeneity observed across venture size and economic sectors, offering a more nuanced understanding of CC awareness and strategic responses.

## Conclusion

This study reveals significant gaps in CC awareness and strategic implementation among MSMEs, yet it also highlights a growing recognition of the direct impact CC has on their operations. By comparing MSMEs with smaller ventures, we provide a nuanced understanding of the varied challenges and opportunities each faces; underscoring the need for targeted educational initiatives, supportive public policies, and strategic partnerships to enhance their capacity to mitigate environmental impact and successfully adapt to CC. Future research should continue to explore these areas, offering deeper insights into effective strategies and interventions that can foster a more sustainable and resilient MSMEs global community. Ultimately, promoting sustainability within MSMEs is not only essential for environmental preservation but also for ensuring long-term economic growth and stability.

## Supporting information

S1 Table Descriptive statistics. (DOCX)

S2 Table Pairwise correlation and variance inflation factor. (DOCX)
