# Supplementary material for: Navigating climate change: Climate change awareness and strategies in micro, small, and medium-sized enterprises in a developing economy
Source: PLoS One. 2025 Jul 2;20(7):e0327165. doi: 10.1371/journal.pone.0327165 (PMC12221047; doi:10.1371/journal.pone.0327165)
Supplement: S1 Table — (DOCX) [file pone.0327165.s001.docx]

Descriptive statistics

| Variable | N | Mean | Median | IQR | Min | Max |
| --- | --- | --- | --- | --- | --- | --- |
| (1) | 1453 | 0.34 | 0.00 | 1.00 | 0.00 | 1.00 |
| (2) | 1453 | 0.40 | 0.00 | 1.00 | 0.00 | 1.00 |
| (3) | 1453 | 0.35 | 0.00 | 1.00 | 0.00 | 1.00 |
| (4) | 1453 | 0.49 | 0.00 | 1.00 | 0.00 | 1.00 |
| (5) | 1453 | 0.31 | 0.00 | 1.00 | 0.00 | 1.00 |
| (6) | 1453 | 0.00 | 0.00 | 0.00 | 0.00 | 1.00 |
| (7) | 1453 | 0.00 | 0.00 | 0.00 | 0.00 | 1.00 |
| (8) | 1453 | 0.74 | 1.00 | 1.00 | 0.00 | 1.00 |
| (9) | 1453 | 0.73 | 1.00 | 1.00 | 0.00 | 1.00 |
| (10) | 1453 | 0.27 | 0.00 | 1.00 | 0.00 | 1.00 |
| (11) | 1453 | 0.11 | 0.00 | 0.00 | 0.00 | 1.00 |
| (12) | 1453 | 0.22 | 0.00 | 0.00 | 0.00 | 1.00 |

Note: (1) Feasibility of achieving carbon neutrality within less than five years; (2) Whether the firm considers carbon neutrality infeasible or does not include it in its strategic plan; (3) Gender-balanced leadership; (4) Capital barriers; (5) Technology barriers; (6) Government support; (7) Stakeholder support-Employees-; (8) Customer pressures; (9) Investor pressures; (10) Awareness of carbon markets; (11) Carbon-related practices; (12) Implementing in carbon programs. N= Size; IQR= Interquartile range; Min= Minimum; Max= Maximum.
