# Supplementary material for: Navigating climate change: Climate change awareness and strategies in micro, small, and medium-sized enterprises in a developing economy
Source: PLoS One. 2025 Jul 2;20(7):e0327165. doi: 10.1371/journal.pone.0327165 (PMC12221047; doi:10.1371/journal.pone.0327165)
Supplement: S2 Table — (DOCX) [file pone.0327165.s002.docx]

Pairwise correlation and variance inflation factor

| Variables | (1) | (2) | (3) | (4) | (5) | (6) | (7) | (8) | (9) | (10) | (11) | (12) | VIFa | VIFb |
| --- | --- | --- | --- | --- | --- | --- | --- | --- | --- | --- | --- | --- | --- | --- |
| (1 | 1.00 |  |  |  |  |  |  |  |  |  |  |  |  |  |
|  |  |  |  |  |  |  |  |  |  |  |  |  |  |  |
| (2) | -0.58 | 1.00 |  |  |  |  |  |  |  |  |  |  |  |  |
|  | (0.00) |  |  |  |  |  |  |  |  |  |  |  |  |  |
| (3) | 0.04 | -0.12 | 1.00 |  |  |  |  |  |  |  |  |  | 1.14 | 1.14 |
|  | (0.09) | (0.00) |  |  |  |  |  |  |  |  |  |  |  |  |
| (4) | 0.05 | -0.13 | 0.01 | 1.00 |  |  |  |  |  |  |  |  | 1.15 | 1.15 |
|  | (0.05) | (0.00) | (0.62) |  |  |  |  |  |  |  |  |  |  |  |
| (5) | 0.00 | -0.08 | 0.03 | 0.34 | 1.00 |  |  |  |  |  |  |  | 1.16 | 1.16 |
|  | (0.94) | (0.00) | (0.34) | (0.00) |  |  |  |  |  |  |  |  |  |  |
| (6) | 0.02 | 0.01 | 0.02 | 0.00 | 0.02 | 1.00 |  |  |  |  |  |  | 1.01 | 1.01 |
|  | (0.49) | (0.67) | (0.53) | (0.98) | (0.40) |  |  |  |  |  |  |  |  |  |
| (7) | -0.02 | 0.01 | -0.02 | 0.00 | -0.02 | 0.00 | 1.00 |  |  |  |  |  | 1.01 | 1.01 |
|  | (0.38) | (0.60) | (0.35) | (0.98) | (0.46) | (0.90) |  |  |  |  |  |  |  |  |
| (8) | 0.09 | -0.15 | 0.04 | 0.05 | 0.08 | 0.00 | -0.04 | 1.00 |  |  |  |  | 1.43 | 1.43 |
|  | (0.00) | (0.00) | (0.16) | (0.05) | (0.00) | (0.96) | (0.18) |  |  |  |  |  |  |  |
| (9) | 0.10 | -0.15 | 0.04 | 0.07 | 0.09 | -0.06 | 0.04 | 0.54 | 1.00 |  |  |  | 1.43 | 1.43 |
|  | (0.00) | (0.00) | (0.17) | (0.00) | (0.00) | (0.03) | (0.14) | (0.00) |  |  |  |  |  |  |
| (10) | 0.09 | -0.14 | 0.14 | 0.09 | 0.02 | 0.00 | 0.01 | 0.04 | 0.07 | 1.00 |  |  | 1.09 | 1.09 |
|  | (0.00) | (0.00) | (0.00) | (0.00) | (0.56) | (0.93) | (0.72) | (0.14) | (0.01) |  |  |  |  |  |
| (11) | 0.06 | -0.13 | 0.05 | 0.04 | 0.09 | -0.02 | -0.02 | 0.05 | 0.00 | 0.20 | 1.00 |  | 1.07 | 1.07 |
|  | (0.02) | (0.00) | (0.06) | (0.09) | (0.00) | (0.49) | (0.39) | (0.04) | (0.87) | (0.00) |  |  |  |  |
| (12) | 0.11 | -0.13 | 0.07 | 0.04 | 0.04 | -0.03 | -0.01 | 0.09 | 0.05 | 0.10 | 0.12 | 1.00 | 1.05 | 1.05 |
|  | (0.00) | (0.00) | (0.01) | (0.17) | (0.12) | (0.29) | (0.75) | (0.00) | (0.07) | (0.00) | (0.00) |  |  |  |

Note: (1) Feasibility of achieving carbon neutrality within less than five years; (2) Whether the firm considers carbon neutrality infeasible or does not include it in its strategic plan; (3) Gender-balanced leadership; (4) Capital barriers; (5) Technology barriers; (6) Government support; (7) Stakeholder support-Employees-; (8) Customer pressures; (9) Investor pressures; (10) Awareness of carbon markets; (11) Carbon-related practices; (12) Implementing in carbon programs.
